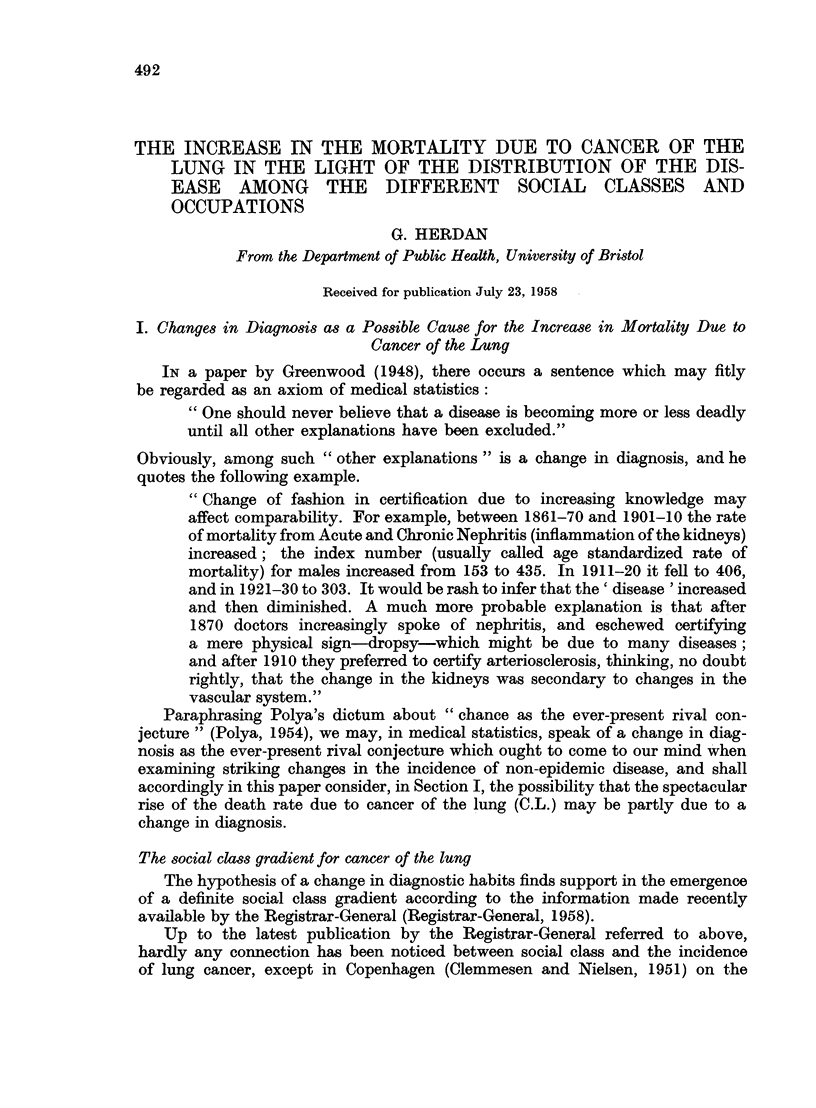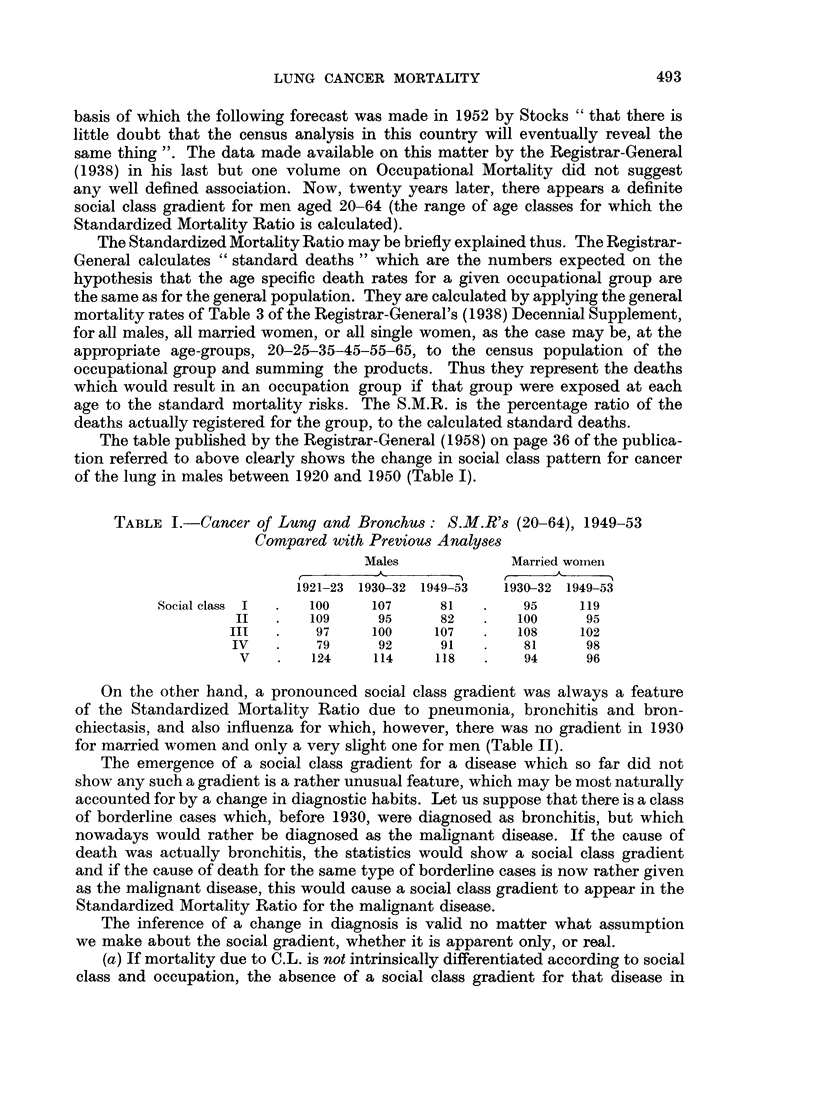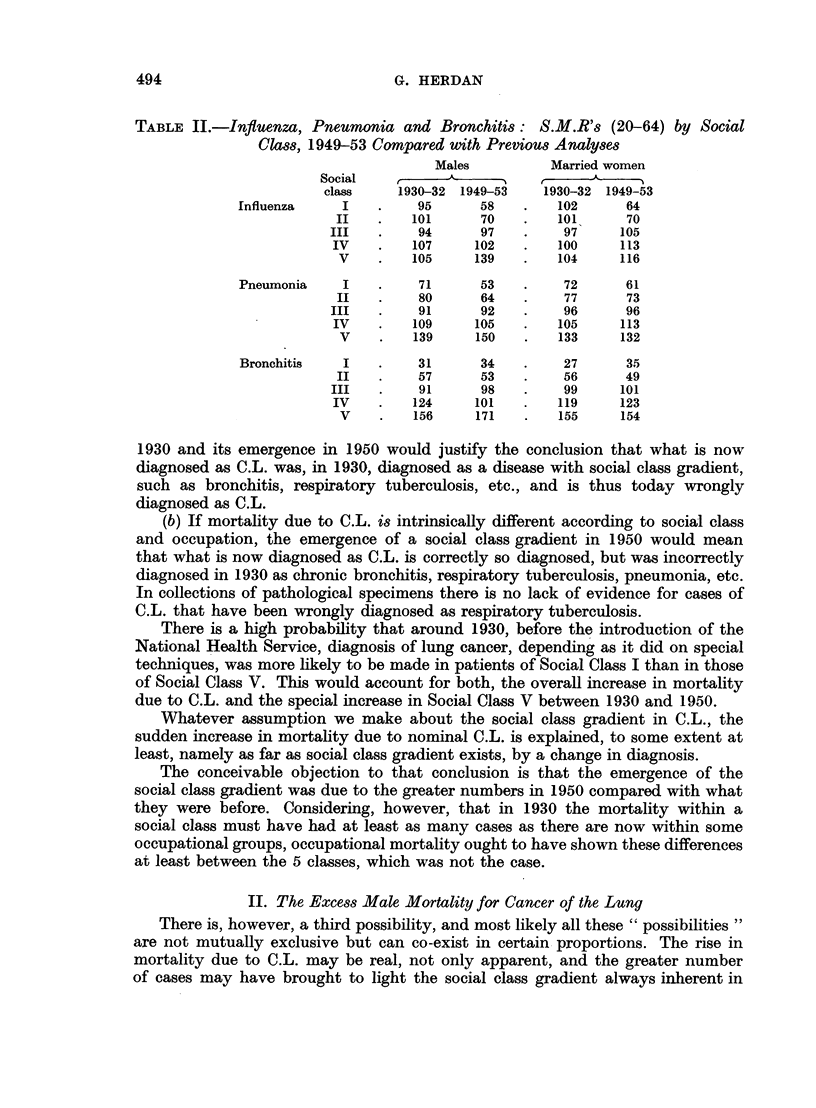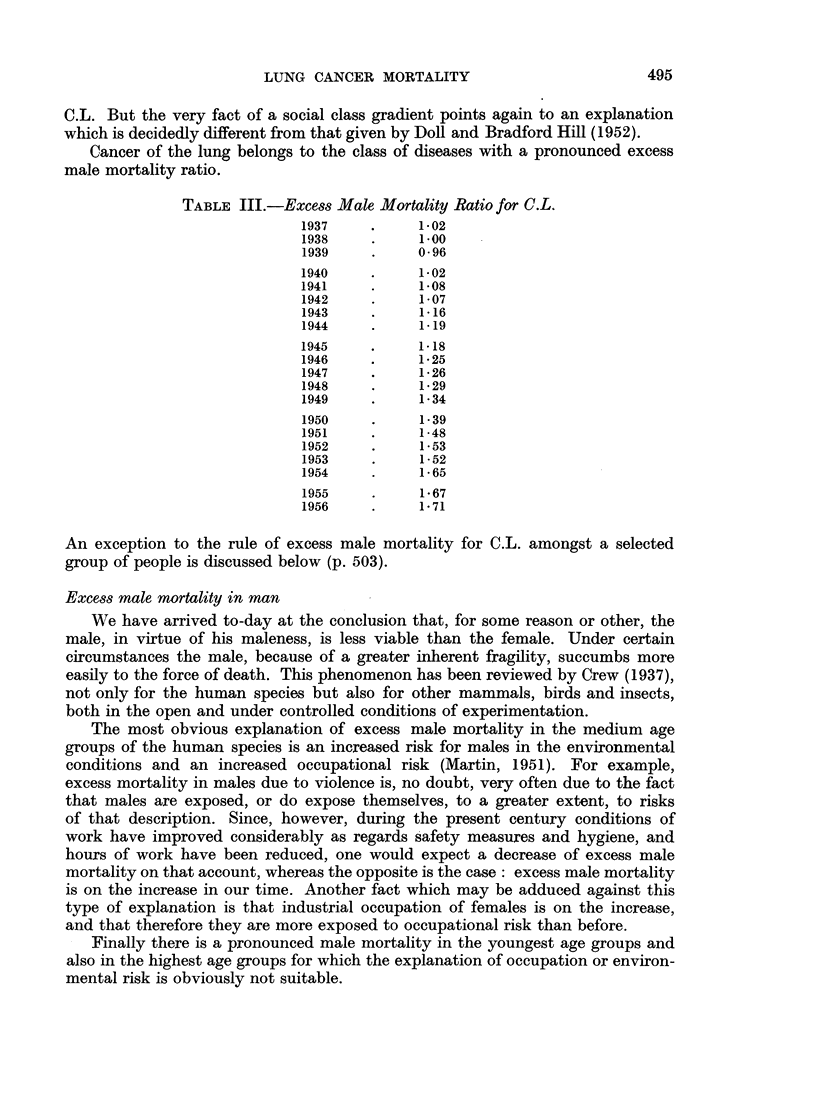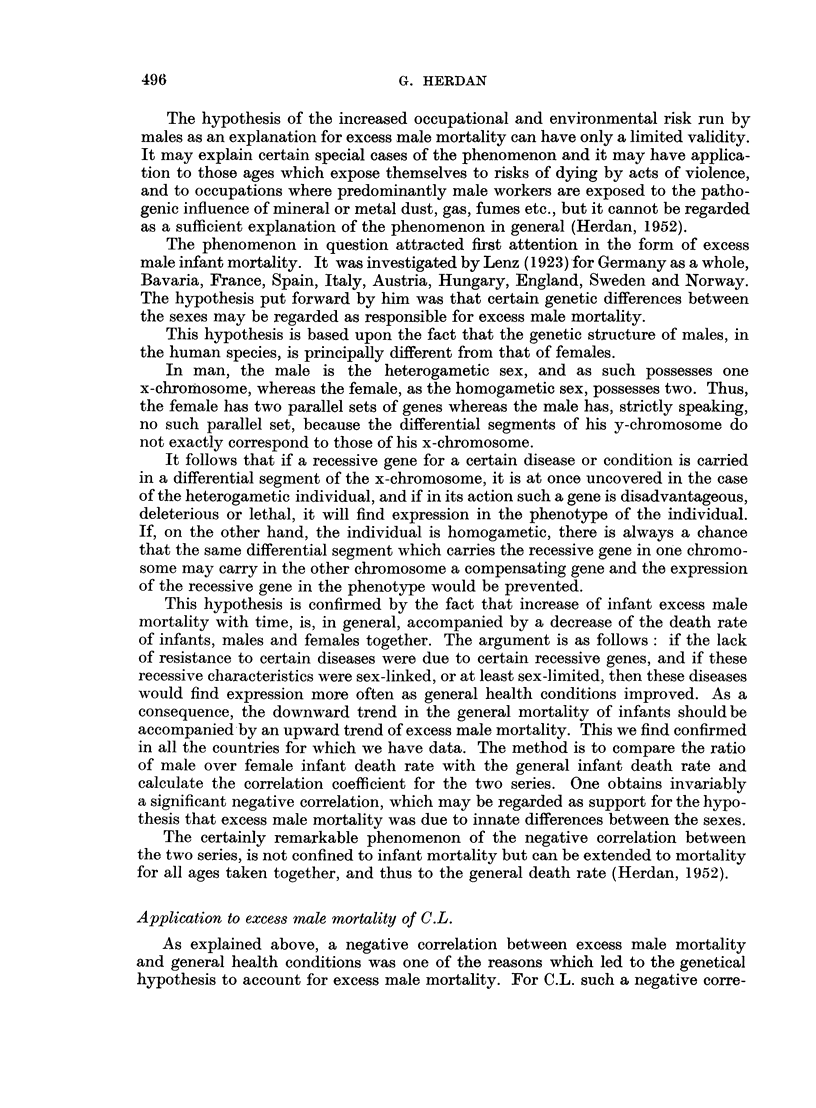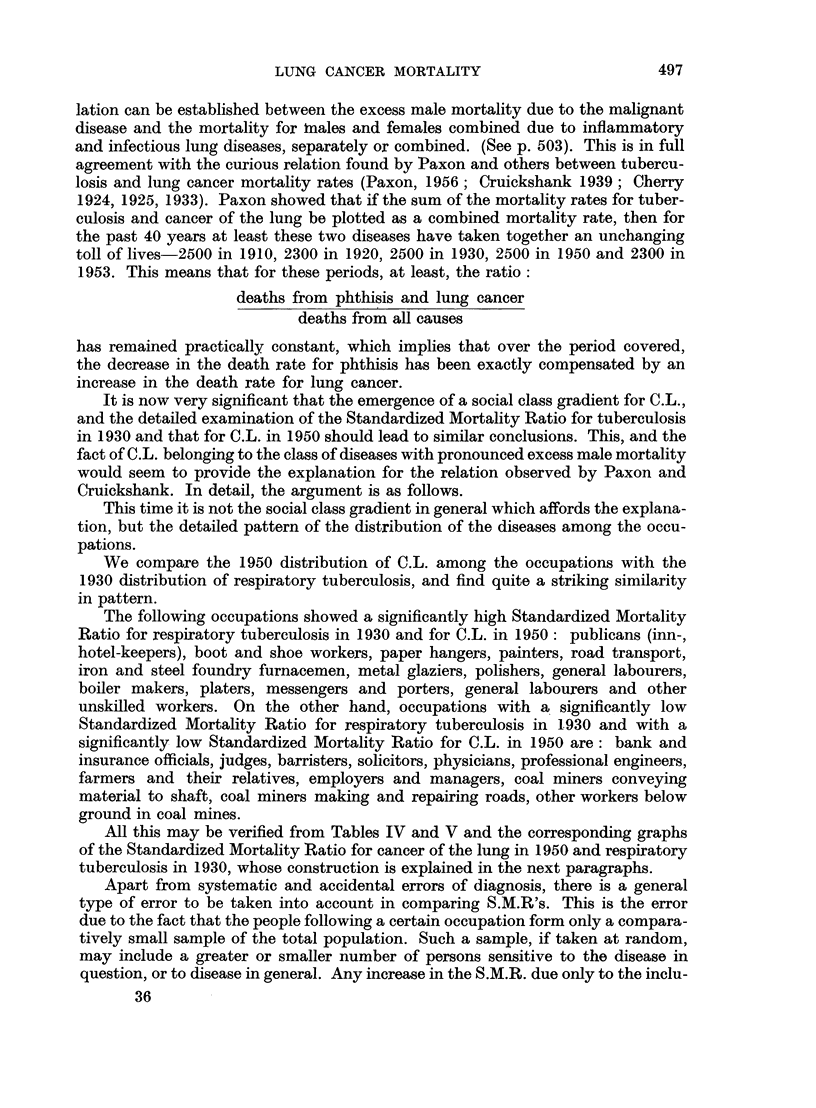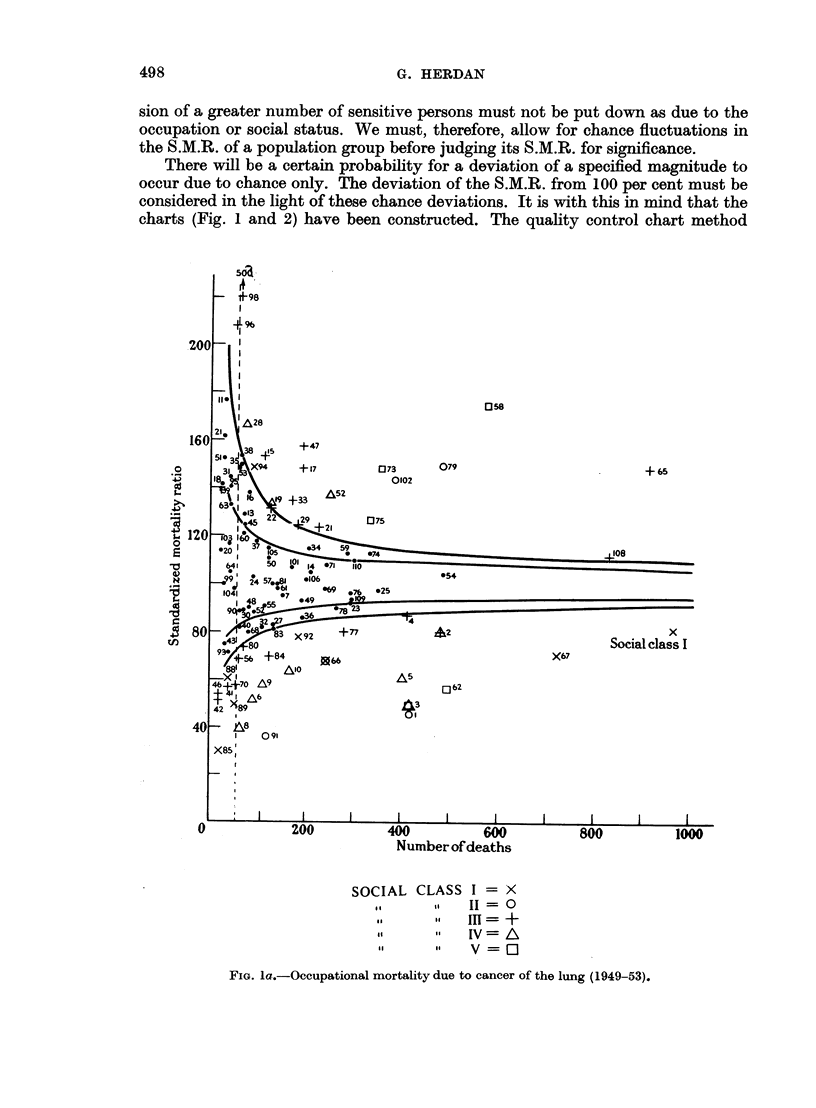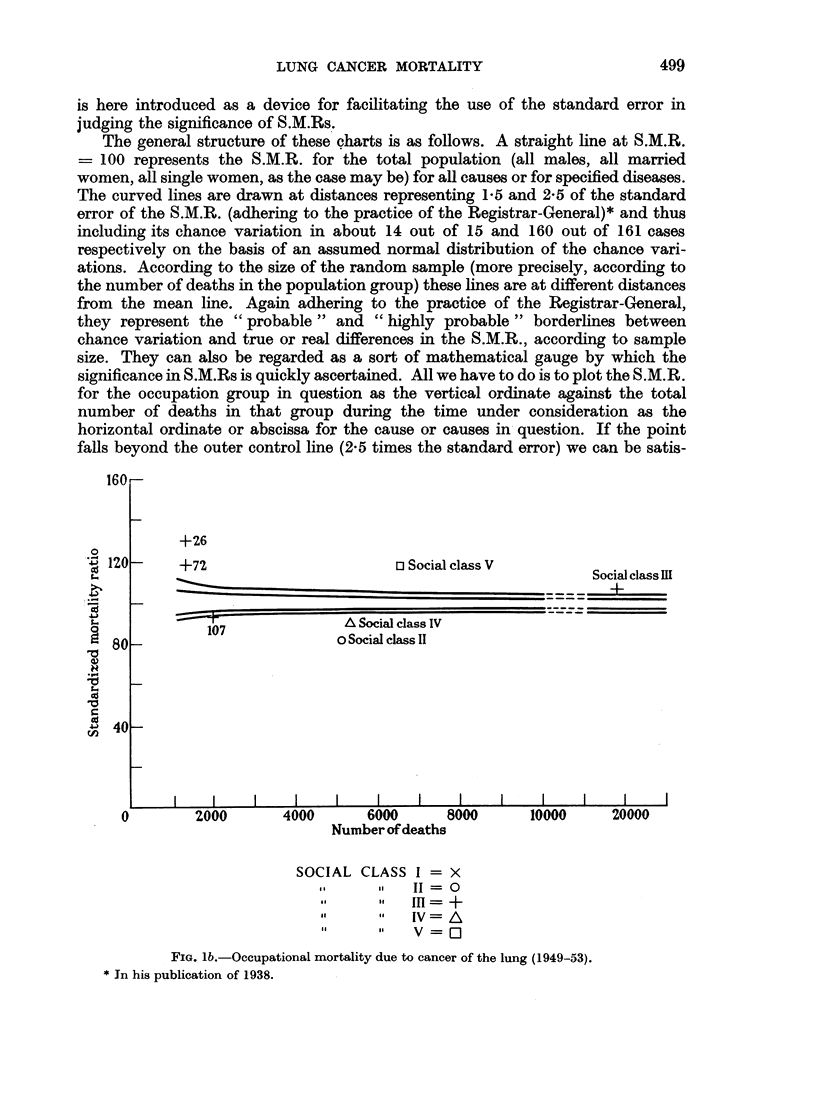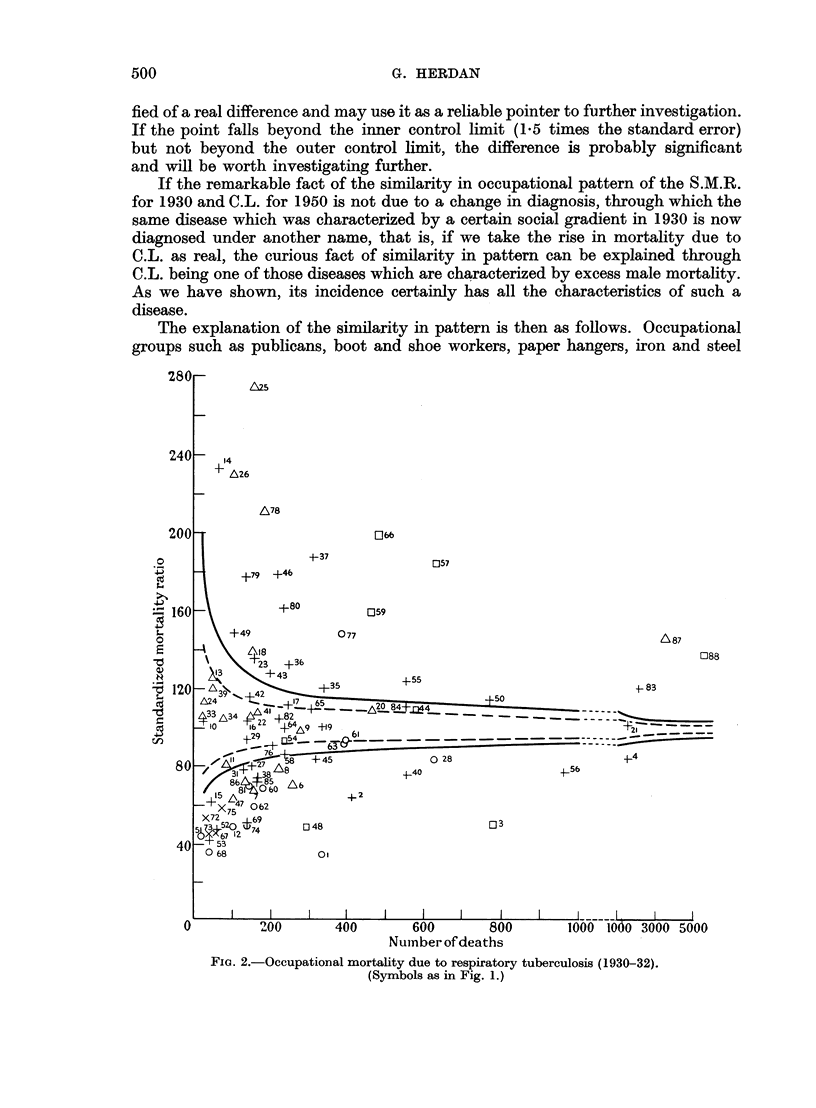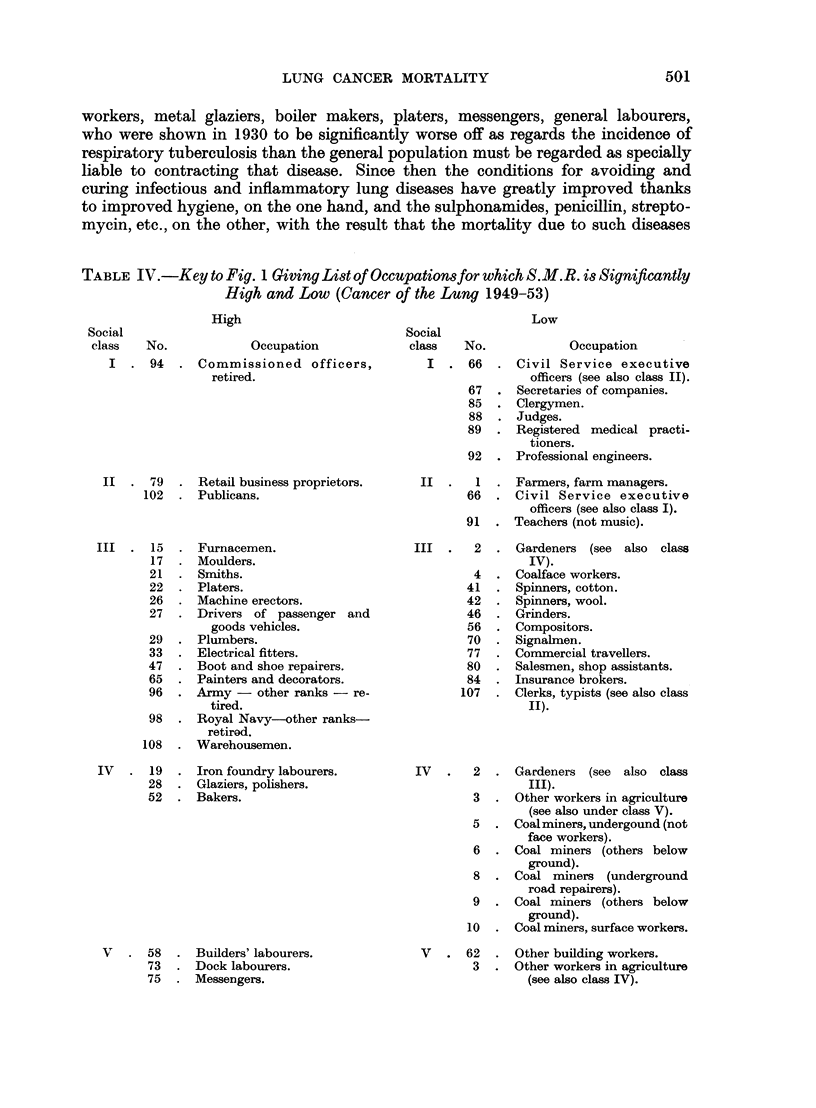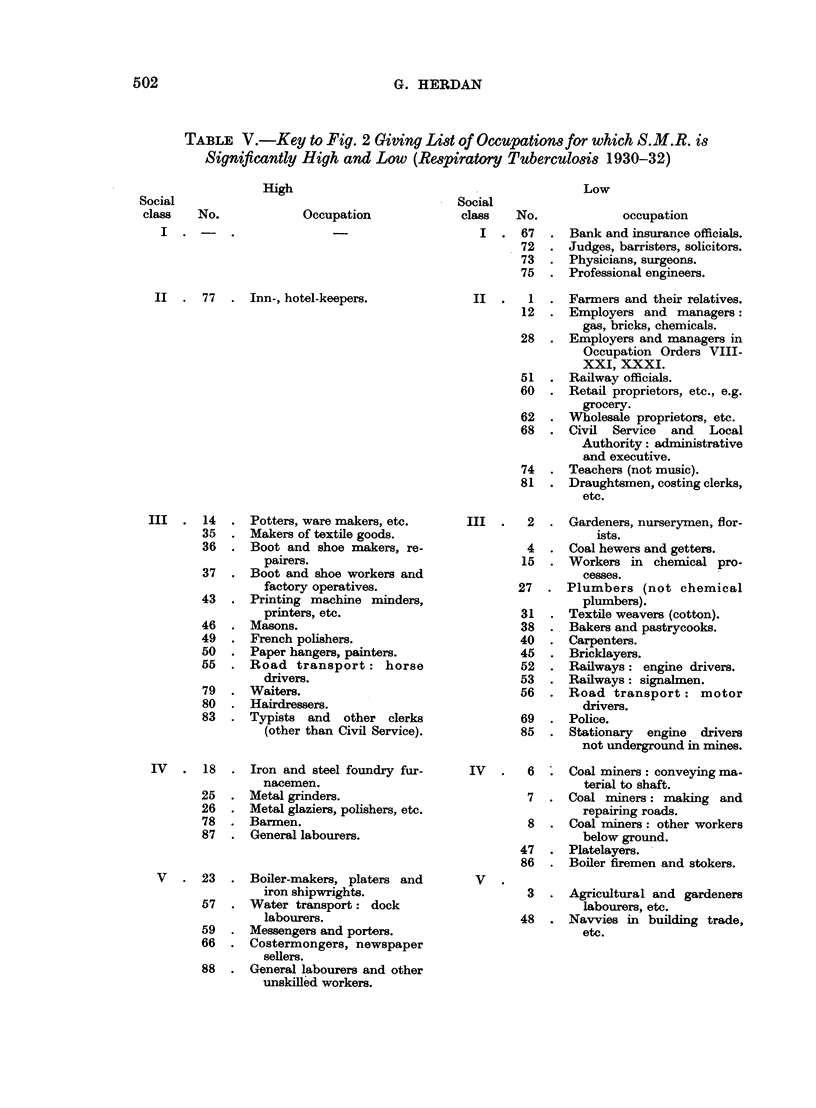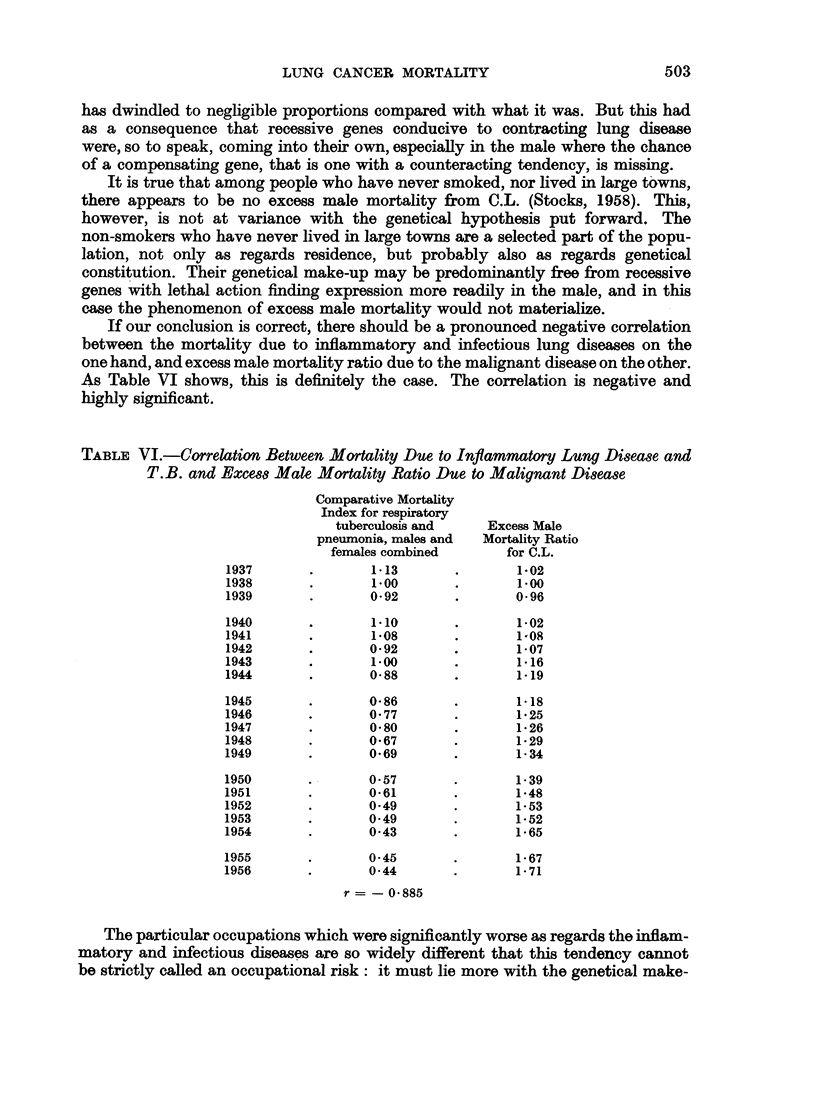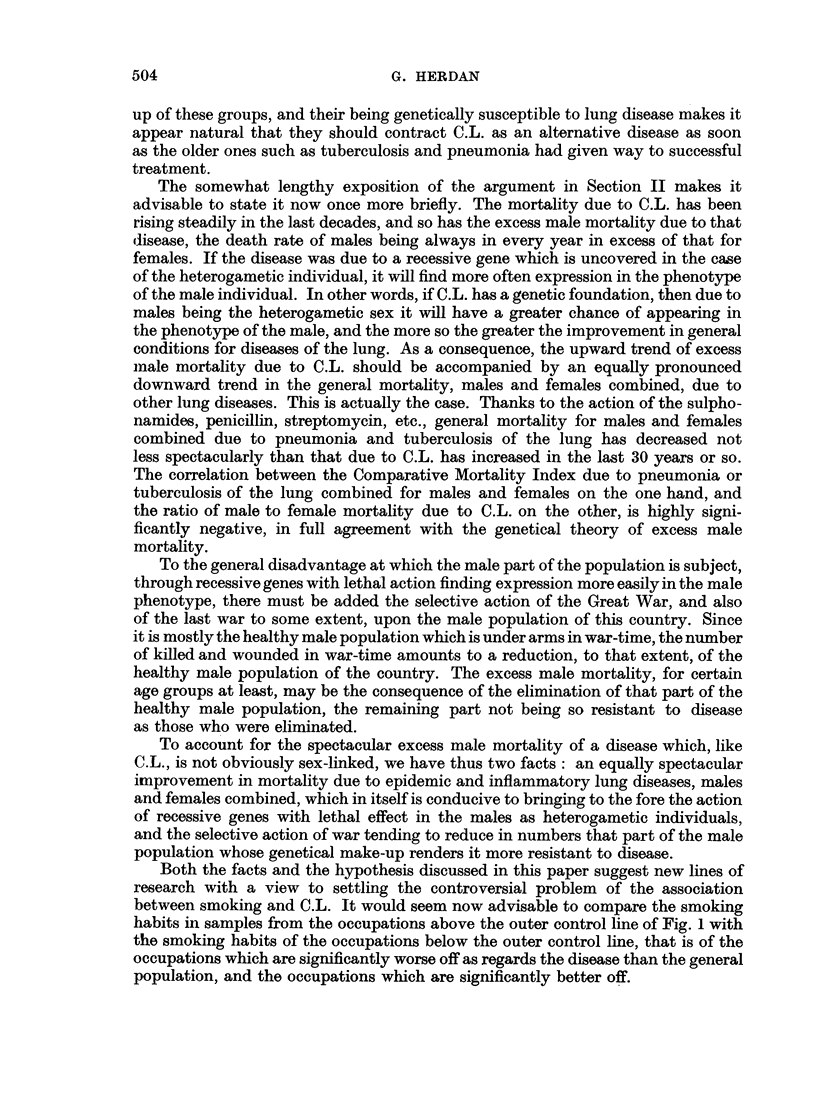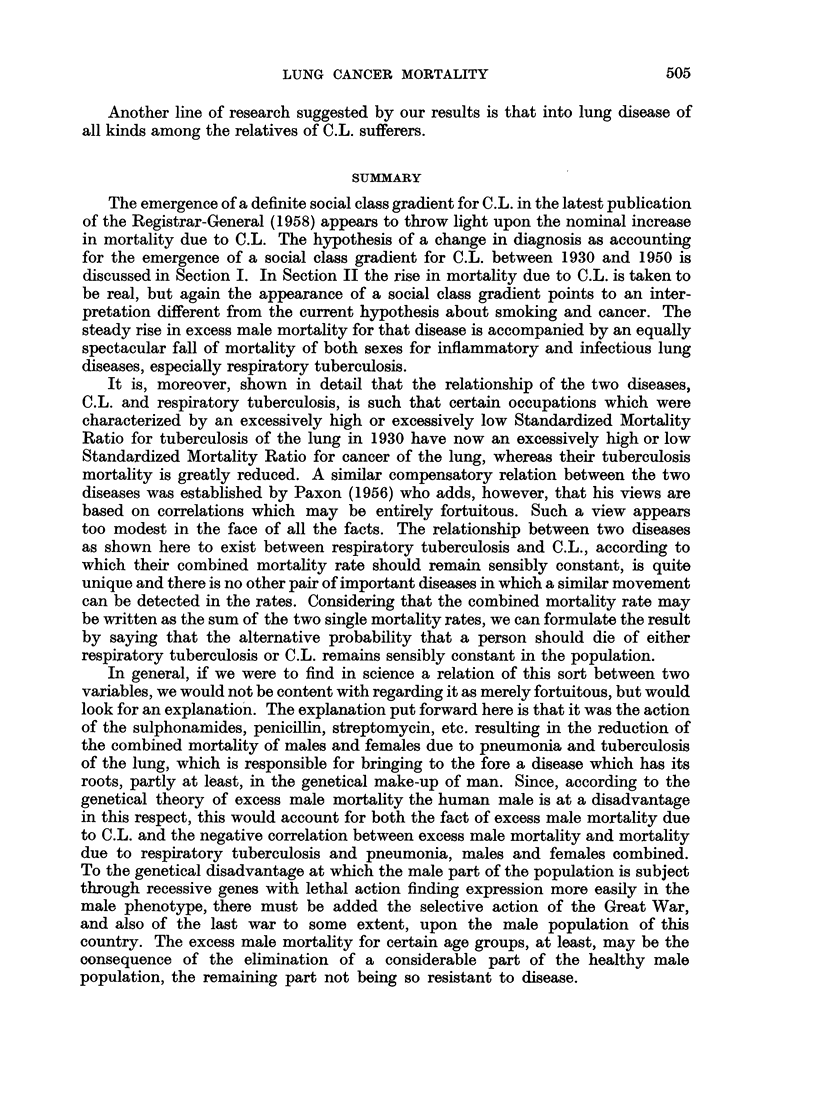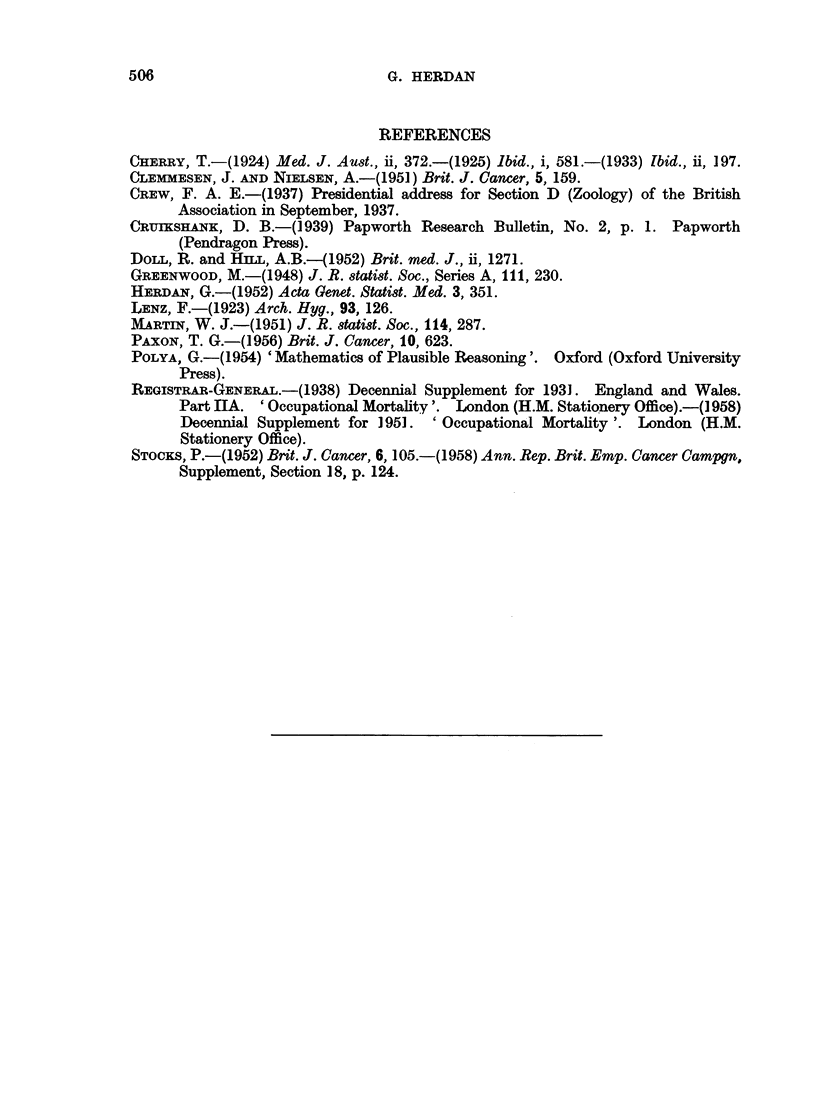# The Increase in the Mortality due to Cancer of the Lung in the Light of the Distribution of the Disease Among the Different Social Classes and Occupations

**DOI:** 10.1038/bjc.1958.57

**Published:** 1958-12

**Authors:** G. Herdan


### THE INCREASE IN THE MORTALITY DUE TO CANCER OF THE LUNG IN THE LIGHT OF THE DISTRIBUTION OF THE DISEASE AMONG THE DIFFERENT SOCIAL CLASSES AND OCCUPATIONS

G. HERDAN

*From the Department of Public Health, University of Bristol*

Received for publication July 23, 1958

### I. *Changes in Diagnosis as a Possible Cause for the Increase in Mortality Due to Cancer of the Lung*

In a paper by Greenwood (1948), there occurs a sentence which may fitly be regarded as an axiom of medical statistics :

> " One should never believe that a disease is becoming more or less deadly until all other explanations have been excluded."

Obviously, among such " other explanations " is a change in diagnosis, and he quotes the following example.

> " Change of fashion in certification due to increasing knowledge may affect comparability. For example, between 1861–70 and 1901–10 the rate of mortality from Acute and Chronic Nephritis (inflammation of the kidneys) increased ; the index number (usually called age standardized rate of mortality) for males increased from 153 to 435. In 1911–20 it fell to 406, and in 1921–30 to 303. It would be rash to infer that the ' disease ' increased and then diminished. A much more probable explanation is that after 1870 doctors increasingly spoke of nephritis, and eschewed certifying a mere physical sign—dropsy—which might be due to many diseases ; and after 1910 they preferred to certify arteriosclerosis, thinking, no doubt rightly, that the change in the kidneys was secondary to changes in the vascular system."

Paraphrasing Polya's dictum about " chance as the ever-present rival conjecture " (Polya, 1954), we may, in medical statistics, speak of a change in diagnosis as the ever-present rival conjecture which ought to come to our mind when examining striking changes in the incidence of non-epidemic disease, and shall accordingly in this paper consider, in Section I, the possibility that the spectacular rise of the death rate due to cancer of the lung (C.L.) may be partly due to a change in diagnosis.

### *The social class gradient for cancer of the lung*

The hypothesis of a change in diagnostic habits finds support in the emergence of a definite social class gradient according to the information made recently available by the Registrar-General (Registrar-General, 1958).

Up to the latest publication by the Registrar-General referred to above, hardly any connection has been noticed between social class and the incidence of lung cancer, except in Copenhagen (Clemmesen and Nielsen, 1951) on the basis of which the following forecast was made in 1952 by Stocks " that there is little doubt that the census analysis in this country will eventually reveal the same thing ". The data made available on this matter by the Registrar-General (1938) in his last but one volume on Occupational Mortality did not suggest any well defined association. Now, twenty years later, there appears a definite social class gradient for men aged 20–64 (the range of age classes for which the Standardized Mortality Ratio is calculated).

The Standardized Mortality Ratio may be briefly explained thus. The Registrar-General calculates " standard deaths " which are the numbers expected on the hypothesis that the age specific death rates for a given occupational group are the same as for the general population. They are calculated by applying the general mortality rates of Table 3 of the Registrar-General's (1938) Decennial Supplement, for all males, all married women, or all single women, as the case may be, at the appropriate age-groups, 20–25–35–45–55–65, to the census population of the occupational group and summing the products. Thus they represent the deaths which would result in an occupation group if that group were exposed at each age to the standard mortality risks. The S.M.R. is the percentage ratio of the deaths actually registered for the group, to the calculated standard deaths.

The table published by the Registrar-General (1958) on page 36 of the publication referred to above clearly shows the change in social class pattern for cancer of the lung in males between 1920 and 1950 (Table I).

TABLE I.—*Cancer of Lung and Bronchus : S.M.R's* (20–64), 1949–53 *Compared with Previous Analyses*

| | Males | | | Married women | |
|---|---|---|---|---|---|
| | 1921–23 | 1930–32 | 1949–53 | 1930–32 | 1949–53 |
| Social class I | 100 | 107 | 81 | 95 | 119 |
| II | 109 | 95 | 82 | 100 | 95 |
| III | 97 | 100 | 107 | 108 | 102 |
| IV | 79 | 92 | 91 | 81 | 98 |
| V | 124 | 114 | 118 | 94 | 96 |

On the other hand, a pronounced social class gradient was always a feature of the Standardized Mortality Ratio due to pneumonia, bronchitis and bronchiectasis, and also influenza for which, however, there was no gradient in 1930 for married women and only a very slight one for men (Table II).

The emergence of a social class gradient for a disease which so far did not show any such a gradient is a rather unusual feature, which may be most naturally accounted for by a change in diagnostic habits. Let us suppose that there is a class of borderline cases which, before 1930, were diagnosed as bronchitis, but which nowadays would rather be diagnosed as the malignant disease. If the cause of death was actually bronchitis, the statistics would show a social class gradient and if the cause of death for the same type of borderline cases is now rather given as the malignant disease, this would cause a social class gradient to appear in the Standardized Mortality Ratio for the malignant disease.

The inference of a change in diagnosis is valid no matter what assumption we make about the social gradient, whether it is apparent only, or real.

(*a*) If mortality due to C.L. is *not* intrinsically differentiated according to social class and occupation, the absence of a social class gradient for that disease in

TABLE II.—*Influenza, Pneumonia and Bronchitis: S.M.R's (20–64) by Social Class, 1949–53 Compared with Previous Analyses*

| | Social class | Males 1930–32 | Males 1949–53 | Married women 1930–32 | Married women 1949–53 |
|---|---|---|---|---|---|
| Influenza | I | 95 | 58 | 102 | 64 |
| | II | 101 | 70 | 101 | 70 |
| | III | 94 | 97 | 97 | 105 |
| | IV | 107 | 102 | 100 | 113 |
| | V | 105 | 139 | 104 | 116 |
| Pneumonia | I | 71 | 53 | 72 | 61 |
| | II | 80 | 64 | 77 | 73 |
| | III | 91 | 92 | 96 | 96 |
| | IV | 109 | 105 | 105 | 113 |
| | V | 139 | 150 | 133 | 132 |
| Bronchitis | I | 31 | 34 | 27 | 35 |
| | II | 57 | 53 | 56 | 49 |
| | III | 91 | 98 | 99 | 101 |
| | IV | 124 | 101 | 119 | 123 |
| | V | 156 | 171 | 155 | 154 |

1930 and its emergence in 1950 would justify the conclusion that what is now diagnosed as C.L. was, in 1930, diagnosed as a disease with social class gradient, such as bronchitis, respiratory tuberculosis, etc., and is thus today wrongly diagnosed as C.L.

(*b*) If mortality due to C.L. *is* intrinsically different according to social class and occupation, the emergence of a social class gradient in 1950 would mean that what is now diagnosed as C.L. is correctly so diagnosed, but was incorrectly diagnosed in 1930 as chronic bronchitis, respiratory tuberculosis, pneumonia, etc. In collections of pathological specimens there is no lack of evidence for cases of C.L. that have been wrongly diagnosed as respiratory tuberculosis.

There is a high probability that around 1930, before the introduction of the National Health Service, diagnosis of lung cancer, depending as it did on special techniques, was more likely to be made in patients of Social Class I than in those of Social Class V. This would account for both, the overall increase in mortality due to C.L. and the special increase in Social Class V between 1930 and 1950.

Whatever assumption we make about the social class gradient in C.L., the sudden increase in mortality due to nominal C.L. is explained, to some extent at least, namely as far as social class gradient exists, by a change in diagnosis.

The conceivable objection to that conclusion is that the emergence of the social class gradient was due to the greater numbers in 1950 compared with what they were before. Considering, however, that in 1930 the mortality within a social class must have had at least as many cases as there are now within some occupational groups, occupational mortality ought to have shown these differences at least between the 5 classes, which was not the case.

### II. *The Excess Male Mortality for Cancer of the Lung*

There is, however, a third possibility, and most likely all these " possibilities " are not mutually exclusive but can co-exist in certain proportions. The rise in mortality due to C.L. may be real, not only apparent, and the greater number of cases may have brought to light the social class gradient always inherent in

C.L. But the very fact of a social class gradient points again to an explanation which is decidedly different from that given by Doll and Bradford Hill (1952).

Cancer of the lung belongs to the class of diseases with a pronounced excess male mortality ratio.

TABLE III.—*Excess Male Mortality Ratio for C.L.*

| | |
|---|---|
| 1937 | 1·02 |
| 1938 | 1·00 |
| 1939 | 0·96 |
| 1940 | 1·02 |
| 1941 | 1·08 |
| 1942 | 1·07 |
| 1943 | 1·16 |
| 1944 | 1·19 |
| 1945 | 1·18 |
| 1946 | 1·25 |
| 1947 | 1·26 |
| 1948 | 1·29 |
| 1949 | 1·34 |
| 1950 | 1·39 |
| 1951 | 1·48 |
| 1952 | 1·53 |
| 1953 | 1·52 |
| 1954 | 1·65 |
| 1955 | 1·67 |
| 1956 | 1·71 |

An exception to the rule of excess male mortality for C.L. amongst a selected group of people is discussed below (p. 503).

*Excess male mortality in man*

We have arrived to-day at the conclusion that, for some reason or other, the male, in virtue of his maleness, is less viable than the female. Under certain circumstances the male, because of a greater inherent fragility, succumbs more easily to the force of death. This phenomenon has been reviewed by Crew (1937), not only for the human species but also for other mammals, birds and insects, both in the open and under controlled conditions of experimentation.

The most obvious explanation of excess male mortality in the medium age groups of the human species is an increased risk for males in the environmental conditions and an increased occupational risk (Martin, 1951). For example, excess mortality in males due to violence is, no doubt, very often due to the fact that males are exposed, or do expose themselves, to a greater extent, to risks of that description. Since, however, during the present century conditions of work have improved considerably as regards safety measures and hygiene, and hours of work have been reduced, one would expect a decrease of excess male mortality on that account, whereas the opposite is the case : excess male mortality is on the increase in our time. Another fact which may be adduced against this type of explanation is that industrial occupation of females is on the increase, and that therefore they are more exposed to occupational risk than before.

Finally there is a pronounced male mortality in the youngest age groups and also in the highest age groups for which the explanation of occupation or environmental risk is obviously not suitable.

The hypothesis of the increased occupational and environmental risk run by males as an explanation for excess male mortality can have only a limited validity. It may explain certain special cases of the phenomenon and it may have application to those ages which expose themselves to risks of dying by acts of violence, and to occupations where predominantly male workers are exposed to the pathogenic influence of mineral or metal dust, gas, fumes etc., but it cannot be regarded as a sufficient explanation of the phenomenon in general (Herdan, 1952).

The phenomenon in question attracted first attention in the form of excess male infant mortality. It was investigated by Lenz (1923) for Germany as a whole, Bavaria, France, Spain, Italy, Austria, Hungary, England, Sweden and Norway. The hypothesis put forward by him was that certain genetic differences between the sexes may be regarded as responsible for excess male mortality.

This hypothesis is based upon the fact that the genetic structure of males, in the human species, is principally different from that of females.

In man, the male is the heterogametic sex, and as such possesses one x-chromosome, whereas the female, as the homogametic sex, possesses two. Thus, the female has two parallel sets of genes whereas the male has, strictly speaking, no such parallel set, because the differential segments of his y-chromosome do not exactly correspond to those of his x-chromosome.

It follows that if a recessive gene for a certain disease or condition is carried in a differential segment of the x-chromosome, it is at once uncovered in the case of the heterogametic individual, and if in its action such a gene is disadvantageous, deleterious or lethal, it will find expression in the phenotype of the individual. If, on the other hand, the individual is homogametic, there is always a chance that the same differential segment which carries the recessive gene in one chromosome may carry in the other chromosome a compensating gene and the expression of the recessive gene in the phenotype would be prevented.

This hypothesis is confirmed by the fact that increase of infant excess male mortality with time, is, in general, accompanied by a decrease of the death rate of infants, males and females together. The argument is as follows : if the lack of resistance to certain diseases were due to certain recessive genes, and if these recessive characteristics were sex-linked, or at least sex-limited, then these diseases would find expression more often as general health conditions improved. As a consequence, the downward trend in the general mortality of infants should be accompanied by an upward trend of excess male mortality. This we find confirmed in all the countries for which we have data. The method is to compare the ratio of male over female infant death rate with the general infant death rate and calculate the correlation coefficient for the two series. One obtains invariably a significant negative correlation, which may be regarded as support for the hypothesis that excess male mortality was due to innate differences between the sexes.

The certainly remarkable phenomenon of the negative correlation between the two series, is not confined to infant mortality but can be extended to mortality for all ages taken together, and thus to the general death rate (Herdan, 1952).

*Application to excess male mortality of C.L.*

As explained above, a negative correlation between excess male mortality and general health conditions was one of the reasons which led to the genetical hypothesis to account for excess male mortality. For C.L. such a negative correlation can be established between the excess male mortality due to the malignant disease and the mortality for males and females combined due to inflammatory and infectious lung diseases, separately or combined. (See p. 503). This is in full agreement with the curious relation found by Paxon and others between tuberculosis and lung cancer mortality rates (Paxon, 1956 ; Cruickshank 1939 ; Cherry 1924, 1925, 1933). Paxon showed that if the sum of the mortality rates for tuberculosis and cancer of the lung be plotted as a combined mortality rate, then for the past 40 years at least these two diseases have taken together an unchanging toll of lives—2500 in 1910, 2300 in 1920, 2500 in 1930, 2500 in 1950 and 2300 in 1953. This means that for these periods, at least, the ratio :

$$\frac{\text{deaths from phthisis and lung cancer}}{\text{deaths from all causes}}$$

has remained practically constant, which implies that over the period covered, the decrease in the death rate for phthisis has been exactly compensated by an increase in the death rate for lung cancer.

It is now very significant that the emergence of a social class gradient for C.L., and the detailed examination of the Standardized Mortality Ratio for tuberculosis in 1930 and that for C.L. in 1950 should lead to similar conclusions. This, and the fact of C.L. belonging to the class of diseases with pronounced excess male mortality would seem to provide the explanation for the relation observed by Paxon and Cruickshank. In detail, the argument is as follows.

This time it is not the social class gradient in general which affords the explanation, but the detailed pattern of the distribution of the diseases among the occupations.

We compare the 1950 distribution of C.L. among the occupations with the 1930 distribution of respiratory tuberculosis, and find quite a striking similarity in pattern.

The following occupations showed a significantly high Standardized Mortality Ratio for respiratory tuberculosis in 1930 and for C.L. in 1950 : publicans (inn-, hotel-keepers), boot and shoe workers, paper hangers, painters, road transport, iron and steel foundry furnacemen, metal glaziers, polishers, general labourers, boiler makers, platers, messengers and porters, general labourers and other unskilled workers. On the other hand, occupations with a significantly low Standardized Mortality Ratio for respiratory tuberculosis in 1930 and with a significantly low Standardized Mortality Ratio for C.L. in 1950 are : bank and insurance officials, judges, barristers, solicitors, physicians, professional engineers, farmers and their relatives, employers and managers, coal miners conveying material to shaft, coal miners making and repairing roads, other workers below ground in coal mines.

All this may be verified from Tables IV and V and the corresponding graphs of the Standardized Mortality Ratio for cancer of the lung in 1950 and respiratory tuberculosis in 1930, whose construction is explained in the next paragraphs.

Apart from systematic and accidental errors of diagnosis, there is a general type of error to be taken into account in comparing S.M.R's. This is the error due to the fact that the people following a certain occupation form only a comparatively small sample of the total population. Such a sample, if taken at random, may include a greater or smaller number of persons sensitive to the disease in question, or to disease in general. Any increase in the S.M.R. due only to the inclusion of a greater number of sensitive persons must not be put down as due to the occupation or social status. We must, therefore, allow for chance fluctuations in the S.M.R. of a population group before judging its S.M.R. for significance.

There will be a certain probability for a deviation of a specified magnitude to occur due to chance only. The deviation of the S.M.R. from 100 per cent must be considered in the light of these chance deviations. It is with this in mind that the charts (Fig. 1 and 2) have been constructed. The quality control chart method

FIG. 1a.—Occupational mortality due to cancer of the lung (1949–53).

is here introduced as a device for facilitating the use of the standard error in judging the significance of S.M.Rs.

The general structure of these charts is as follows. A straight line at S.M.R. = 100 represents the S.M.R. for the total population (all males, all married women, all single women, as the case may be) for all causes or for specified diseases. The curved lines are drawn at distances representing 1·5 and 2·5 of the standard error of the S.M.R. (adhering to the practice of the Registrar-General)* and thus including its chance variation in about 14 out of 15 and 160 out of 161 cases respectively on the basis of an assumed normal distribution of the chance variations. According to the size of the random sample (more precisely, according to the number of deaths in the population group) these lines are at different distances from the mean line. Again adhering to the practice of the Registrar-General, they represent the " probable " and " highly probable " borderlines between chance variation and true or real differences in the S.M.R., according to sample size. They can also be regarded as a sort of mathematical gauge by which the significance in S.M.Rs is quickly ascertained. All we have to do is to plot the S.M.R. for the occupation group in question as the vertical ordinate against the total number of deaths in that group during the time under consideration as the horizontal ordinate or abscissa for the cause or causes in question. If the point falls beyond the outer control line (2·5 times the standard error) we can be satis-

SOCIAL CLASS I = ×
" " II = ○
" " III = +
" " IV = △
" " V = □

FIG. 1*b*.—Occupational mortality due to cancer of the lung (1949–53).

* In his publication of 1938.

fied of a real difference and may use it as a reliable pointer to further investigation. If the point falls beyond the inner control limit (1·5 times the standard error) but not beyond the outer control limit, the difference is probably significant and will be worth investigating further.

If the remarkable fact of the similarity in occupational pattern of the S.M.R. for 1930 and C.L. for 1950 is not due to a change in diagnosis, through which the same disease which was characterized by a certain social gradient in 1930 is now diagnosed under another name, that is, if we take the rise in mortality due to C.L. as real, the curious fact of similarity in pattern can be explained through C.L. being one of those diseases which are characterized by excess male mortality. As we have shown, its incidence certainly has all the characteristics of such a disease.

The explanation of the similarity in pattern is then as follows. Occupational groups such as publicans, boot and shoe workers, paper hangers, iron and steel

FIG. 2.—Occupational mortality due to respiratory tuberculosis (1930–32). (Symbols as in Fig. 1.)

workers, metal glaziers, boiler makers, platers, messengers, general labourers, who were shown in 1930 to be significantly worse off as regards the incidence of respiratory tuberculosis than the general population must be regarded as specially liable to contracting that disease. Since then the conditions for avoiding and curing infectious and inflammatory lung diseases have greatly improved thanks to improved hygiene, on the one hand, and the sulphonamides, penicillin, streptomycin, etc., on the other, with the result that the mortality due to such diseases

TABLE IV.—*Key to Fig. 1 Giving List of Occupations for which S.M.R. is Significantly High and Low (Cancer of the Lung 1949–53)*

| High | | | Low | | |
|---|---|---|---|---|---|
| Social class | No. | Occupation | Social class | No. | Occupation |
| I | 94 | Commissioned officers, retired. | I | 66 | Civil Service executive officers (see also class II). |
| | | | | 67 | Secretaries of companies. |
| | | | | 85 | Clergymen. |
| | | | | 88 | Judges. |
| | | | | 89 | Registered medical practitioners. |
| | | | | 92 | Professional engineers. |
| II | 79 | Retail business proprietors. | II | 1 | Farmers, farm managers. |
| | 102 | Publicans. | | 66 | Civil Service executive officers (see also class I). |
| | | | | 91 | Teachers (not music). |
| III | 15 | Furnacemen. | III | 2 | Gardeners (see also class IV). |
| | 17 | Moulders. | | 4 | Coalface workers. |
| | 21 | Smiths. | | 41 | Spinners, cotton. |
| | 22 | Platers. | | 42 | Spinners, wool. |
| | 26 | Machine erectors. | | 46 | Grinders. |
| | 27 | Drivers of passenger and goods vehicles. | | 56 | Compositors. |
| | 29 | Plumbers. | | 70 | Signalmen. |
| | 33 | Electrical fitters. | | 77 | Commercial travellers. |
| | 47 | Boot and shoe repairers. | | 80 | Salesmen, shop assistants. |
| | 65 | Painters and decorators. | | 84 | Insurance brokers. |
| | 96 | Army — other ranks — retired. | | 107 | Clerks, typists (see also class II). |
| | 98 | Royal Navy—other ranks—retired. | | | |
| | 108 | Warehousemen. | | | |
| IV | 19 | Iron foundry labourers. | IV | 2 | Gardeners (see also class III). |
| | 28 | Glaziers, polishers. | | 3 | Other workers in agriculture (see also under class V). |
| | 52 | Bakers. | | 5 | Coal miners, undergound (not face workers). |
| | | | | 6 | Coal miners (others below ground). |
| | | | | 8 | Coal miners (underground road repairers). |
| | | | | 9 | Coal miners (others below ground). |
| | | | | 10 | Coal miners, surface workers. |
| V | 58 | Builders' labourers. | V | 62 | Other building workers. |
| | 73 | Dock labourers. | | 3 | Other workers in agriculture (see also class IV). |
| | 75 | Messengers. | | | |

TABLE V.—*Key to Fig. 2 Giving List of Occupations for which S.M.R. is Significantly High and Low (Respiratory Tuberculosis* 1930–32)

**High**

| Social class | No. | Occupation |
|---|---|---|
| I | — | — |
| II | 77 | Inn-, hotel-keepers. |
| III | 14 | Potters, ware makers, etc. |
| | 35 | Makers of textile goods. |
| | 36 | Boot and shoe makers, repairers. |
| | 37 | Boot and shoe workers and factory operatives. |
| | 43 | Printing machine minders, printers, etc. |
| | 46 | Masons. |
| | 49 | French polishers. |
| | 50 | Paper hangers, painters. |
| | 55 | Road transport: horse drivers. |
| | 79 | Waiters. |
| | 80 | Hairdressers. |
| | 83 | Typists and other clerks (other than Civil Service). |
| IV | 18 | Iron and steel foundry furnacemen. |
| | 25 | Metal grinders. |
| | 26 | Metal glaziers, polishers, etc. |
| | 78 | Barmen. |
| | 87 | General labourers. |
| V | 23 | Boiler-makers, platers and iron shipwrights. |
| | 57 | Water transport: dock labourers. |
| | 59 | Messengers and porters. |
| | 66 | Costermongers, newspaper sellers. |
| | 88 | General labourers and other unskilled workers. |

**Low**

| Social class | No. | occupation |
|---|---|---|
| I | 67 | Bank and insurance officials. |
| | 72 | Judges, barristers, solicitors. |
| | 73 | Physicians, surgeons. |
| | 75 | Professional engineers. |
| II | 1 | Farmers and their relatives. |
| | 12 | Employers and managers: gas, bricks, chemicals. |
| | 28 | Employers and managers in Occupation Orders VIII-XXI, XXXI. |
| | 51 | Railway officials. |
| | 60 | Retail proprietors, etc., e.g. grocery. |
| | 62 | Wholesale proprietors, etc. |
| | 68 | Civil Service and Local Authority: administrative and executive. |
| | 74 | Teachers (not music). |
| | 81 | Draughtsmen, costing clerks, etc. |
| III | 2 | Gardeners, nurserymen, florists. |
| | 4 | Coal hewers and getters. |
| | 15 | Workers in chemical processes. |
| | 27 | Plumbers (not chemical plumbers). |
| | 31 | Textile weavers (cotton). |
| | 38 | Bakers and pastrycooks. |
| | 40 | Carpenters. |
| | 45 | Bricklayers. |
| | 52 | Railways: engine drivers. |
| | 53 | Railways: signalmen. |
| | 56 | Road transport: motor drivers. |
| | 69 | Police. |
| | 85 | Stationary engine drivers not underground in mines. |
| IV | 6 | Coal miners: conveying material to shaft. |
| | 7 | Coal miners: making and repairing roads. |
| | 8 | Coal miners: other workers below ground. |
| | 47 | Platelayers. |
| | 86 | Boiler firemen and stokers. |
| V | 3 | Agricultural and gardeners labourers, etc. |
| | 48 | Navvies in building trade, etc. |

has dwindled to negligible proportions compared with what it was. But this had as a consequence that recessive genes conducive to contracting lung disease were, so to speak, coming into their own, especially in the male where the chance of a compensating gene, that is one with a counteracting tendency, is missing.

It is true that among people who have never smoked, nor lived in large towns, there appears to be no excess male mortality from C.L. (Stocks, 1958). This, however, is not at variance with the genetical hypothesis put forward. The non-smokers who have never lived in large towns are a selected part of the population, not only as regards residence, but probably also as regards genetical constitution. Their genetical make-up may be predominantly free from recessive genes with lethal action finding expression more readily in the male, and in this case the phenomenon of excess male mortality would not materialize.

If our conclusion is correct, there should be a pronounced negative correlation between the mortality due to inflammatory and infectious lung diseases on the one hand, and excess male mortality ratio due to the malignant disease on the other. As Table VI shows, this is definitely the case. The correlation is negative and highly significant.

TABLE VI.—*Correlation Between Mortality Due to Inflammatory Lung Disease and T.B. and Excess Male Mortality Ratio Due to Malignant Disease*

| | Comparative Mortality Index for respiratory tuberculosis and pneumonia, males and females combined | Excess Male Mortality Ratio for C.L. |
|---|---|---|
| 1937 | 1·13 | 1·02 |
| 1938 | 1·00 | 1·00 |
| 1939 | 0·92 | 0·96 |
| 1940 | 1·10 | 1·02 |
| 1941 | 1·08 | 1·08 |
| 1942 | 0·92 | 1·07 |
| 1943 | 1·00 | 1·16 |
| 1944 | 0·88 | 1·19 |
| 1945 | 0·86 | 1·18 |
| 1946 | 0·77 | 1·25 |
| 1947 | 0·80 | 1·26 |
| 1948 | 0·67 | 1·29 |
| 1949 | 0·69 | 1·34 |
| 1950 | 0·57 | 1·39 |
| 1951 | 0·61 | 1·48 |
| 1952 | 0·49 | 1·53 |
| 1953 | 0·49 | 1·52 |
| 1954 | 0·43 | 1·65 |
| 1955 | 0·45 | 1·67 |
| 1956 | 0·44 | 1·71 |

$r = -0{\cdot}885$

The particular occupations which were significantly worse as regards the inflammatory and infectious diseases are so widely different that this tendency cannot be strictly called an occupational risk : it must lie more with the genetical makeup of these groups, and their being genetically susceptible to lung disease makes it appear natural that they should contract C.L. as an alternative disease as soon as the older ones such as tuberculosis and pneumonia had given way to successful treatment.

The somewhat lengthy exposition of the argument in Section II makes it advisable to state it now once more briefly. The mortality due to C.L. has been rising steadily in the last decades, and so has the excess male mortality due to that disease, the death rate of males being always in every year in excess of that for females. If the disease was due to a recessive gene which is uncovered in the case of the heterogametic individual, it will find more often expression in the phenotype of the male individual. In other words, if C.L. has a genetic foundation, then due to males being the heterogametic sex it will have a greater chance of appearing in the phenotype of the male, and the more so the greater the improvement in general conditions for diseases of the lung. As a consequence, the upward trend of excess male mortality due to C.L. should be accompanied by an equally pronounced downward trend in the general mortality, males and females combined, due to other lung diseases. This is actually the case. Thanks to the action of the sulphonamides, penicillin, streptomycin, etc., general mortality for males and females combined due to pneumonia and tuberculosis of the lung has decreased not less spectacularly than that due to C.L. has increased in the last 30 years or so. The correlation between the Comparative Mortality Index due to pneumonia or tuberculosis of the lung combined for males and females on the one hand, and the ratio of male to female mortality due to C.L. on the other, is highly significantly negative, in full agreement with the genetical theory of excess male mortality.

To the general disadvantage at which the male part of the population is subject, through recessive genes with lethal action finding expression more easily in the male phenotype, there must be added the selective action of the Great War, and also of the last war to some extent, upon the male population of this country. Since it is mostly the healthy male population which is under arms in war-time, the number of killed and wounded in war-time amounts to a reduction, to that extent, of the healthy male population of the country. The excess male mortality, for certain age groups at least, may be the consequence of the elimination of that part of the healthy male population, the remaining part not being so resistant to disease as those who were eliminated.

To account for the spectacular excess male mortality of a disease which, like C.L., is not obviously sex-linked, we have thus two facts : an equally spectacular improvement in mortality due to epidemic and inflammatory lung diseases, males and females combined, which in itself is conducive to bringing to the fore the action of recessive genes with lethal effect in the males as heterogametic individuals, and the selective action of war tending to reduce in numbers that part of the male population whose genetical make-up renders it more resistant to disease.

Both the facts and the hypothesis discussed in this paper suggest new lines of research with a view to settling the controversial problem of the association between smoking and C.L. It would seem now advisable to compare the smoking habits in samples from the occupations above the outer control line of Fig. 1 with the smoking habits of the occupations below the outer control line, that is of the occupations which are significantly worse off as regards the disease than the general population, and the occupations which are significantly better off.

Another line of research suggested by our results is that into lung disease of all kinds among the relatives of C.L. sufferers.

### SUMMARY

The emergence of a definite social class gradient for C.L. in the latest publication of the Registrar-General (1958) appears to throw light upon the nominal increase in mortality due to C.L. The hypothesis of a change in diagnosis as accounting for the emergence of a social class gradient for C.L. between 1930 and 1950 is discussed in Section I. In Section II the rise in mortality due to C.L. is taken to be real, but again the appearance of a social class gradient points to an interpretation different from the current hypothesis about smoking and cancer. The steady rise in excess male mortality for that disease is accompanied by an equally spectacular fall of mortality of both sexes for inflammatory and infectious lung diseases, especially respiratory tuberculosis.

It is, moreover, shown in detail that the relationship of the two diseases, C.L. and respiratory tuberculosis, is such that certain occupations which were characterized by an excessively high or excessively low Standardized Mortality Ratio for tuberculosis of the lung in 1930 have now an excessively high or low Standardized Mortality Ratio for cancer of the lung, whereas their tuberculosis mortality is greatly reduced. A similar compensatory relation between the two diseases was established by Paxon (1956) who adds, however, that his views are based on correlations which may be entirely fortuitous. Such a view appears too modest in the face of all the facts. The relationship between two diseases as shown here to exist between respiratory tuberculosis and C.L., according to which their combined mortality rate should remain sensibly constant, is quite unique and there is no other pair of important diseases in which a similar movement can be detected in the rates. Considering that the combined mortality rate may be written as the sum of the two single mortality rates, we can formulate the result by saying that the alternative probability that a person should die of either respiratory tuberculosis or C.L. remains sensibly constant in the population.

In general, if we were to find in science a relation of this sort between two variables, we would not be content with regarding it as merely fortuitous, but would look for an explanation. The explanation put forward here is that it was the action of the sulphonamides, penicillin, streptomycin, etc. resulting in the reduction of the combined mortality of males and females due to pneumonia and tuberculosis of the lung, which is responsible for bringing to the fore a disease which has its roots, partly at least, in the genetical make-up of man. Since, according to the genetical theory of excess male mortality the human male is at a disadvantage in this respect, this would account for both the fact of excess male mortality due to C.L. and the negative correlation between excess male mortality and mortality due to respiratory tuberculosis and pneumonia, males and females combined. To the genetical disadvantage at which the male part of the population is subject through recessive genes with lethal action finding expression more easily in the male phenotype, there must be added the selective action of the Great War, and also of the last war to some extent, upon the male population of this country. The excess male mortality for certain age groups, at least, may be the consequence of the elimination of a considerable part of the healthy male population, the remaining part not being so resistant to disease.

### REFERENCES

CHERRY, T.—(1924) *Med. J. Aust.*, ii, 372.—(1925) *Ibid.*, i, 581.—(1933) *Ibid.*, ii, 197.

CLEMMESEN, J. AND NIELSEN, A.—(1951) *Brit. J. Cancer*, **5**, 159.

CREW, F. A. E.—(1937) Presidential address for Section D (Zoology) of the British Association in September, 1937.

CRUIKSHANK, D. B.—(1939) Papworth Research Bulletin, No. 2, p. 1. Papworth (Pendragon Press).

DOLL, R. and HILL, A.B.—(1952) *Brit. med. J.*, ii, 1271.

GREENWOOD, M.—(1948) *J. R. statist. Soc.*, Series A, **111**, 230.

HERDAN, G.—(1952) *Acta Genet. Statist. Med.* **3**, 351.

LENZ, F.—(1923) *Arch. Hyg.*, **93**, 126.

MARTIN, W. J.—(1951) *J. R. statist. Soc.*, **114**, 287.

PAXON, T. G.—(1956) *Brit. J. Cancer*, **10**, 623.

POLYA, G.—(1954) 'Mathematics of Plausible Reasoning'. Oxford (Oxford University Press).

REGISTRAR-GENERAL.—(1938) Decennial Supplement for 1931. England and Wales. Part IIA. 'Occupational Mortality'. London (H.M. Stationery Office).—(1958) Decennial Supplement for 1951. 'Occupational Mortality'. London (H.M. Stationery Office).

STOCKS, P.—(1952) *Brit. J. Cancer*, **6**, 105.—(1958) *Ann. Rep. Brit. Emp. Cancer Campgn*, Supplement, Section 18, p. 124.